# Recent advances on protein engineering for improved stability

**DOI:** 10.1016/j.bidere.2025.100005

**Published:** 2025-02-26

**Authors:** Jinghao Shi, Bo Yuan, Hengquan Yang, Zhoutong Sun

**Affiliations:** a School of Chemistry and Chemical Engineering, Shanxi University, 030006, Taiyuan, China; b Tianjin Institute of Industrial Biotechnology, Chinese Academy of Sciences, 32 West 7th Avenue, Tianjin Airport Economic Area, Tianjin, 300308, China; c Key Laboratory of Engineering Biology for Low-Carbon Manufacturing, Tianjin, 300308, China; d Shanxi Key Laboratory of the Green Catalytic Synthesis of Coal-based High Value Chemicals, China

**Keywords:** Thermostability, Biocatalysis, Protein engineering, Directed evolution, B-factor

## Abstract

Stability is a crucial factor influencing applicability of industrial biocatalysts, and enzymatic operations in partial organic solvents and higher temperatures are preferable since organic solvents may improve the solubility of the substrates, and the reaction rates are often higher at elevated temperatures. This review aims to summarize recent advances for engineering enzymes to meet the industrial needs for solvent and thermostability and offer insights in the advances in methodologies utilizing B-factors, ancestral reconstructions, or machine learning approaches.

## 1 Introduction

### 1.1 A short historical outline for protein engineering - from random mutagenesis to (semi) rational design

To mimic the Darwinian evolution in nature, since 1960s, scientists have strived to develop a reliable methodology. Since the molecular biological technique was established by Michael Smith in the late 1970s [1] and polymerase chain reaction (PCR) for DNA amplification, which led to Nobel Prizes [2,3], applications of the techniques have been rapidly expanded.

In 1967, the first in-vitro evolution experiment was reported by Spiegelmann et al., and the term ‘directed evolution’ was firstly used by Fancis and Hansche [4], describing a system involving *Saccharomyces cerevisiae* and an acid phosphatase. In vivo directed evolution processes were described later in the 1970s, where the Hall group explored new functions of ebgA (evolved β-galactosidase) [5]. Recently Liu et al. developed the phage-assisted continuous evolution (PACE) technique, which could be viewed as extensions of the evolution methods [6,7]. The first formulation of non-nature Darwinian evolution by gene mutagenesis, amplification and selection was described in 1984 by Eigen and Gardiner et al. [8].

Following the advancements in the ‘megaprimer’ method reported in 1989 [9], major developments were reported and later led to the QuikChange™ protocol for saturation mutagenesis in 2002 [10,11]. The first error-prone polymerase chain reaction (epPCR) was described by Goeddel et al., in 1989, however, it did not find important applications till 1992 by Hawkins and Winter, who reported the creation of combinatorial libraries of antibodies [12]. In 1993, Chen and Arnold reported the use of epPCR in the random mutagenesis of a protease subtilisin E to enhance the organic solvent (DMF) resistance, which was highlighted during the Nobel Prize lecture by Frances H. Arnold in 2018. A few years later, DNA shuffling, as another innovative random mutagenesis method, was reported by Stemmer in 1994 [13,14]. Following the initial success in directed evolution, research interests were sparkled in the 20th century.

The general procedures for enzyme engineering include the gene diversification and screening or selection steps. Till now, the non-rational methods for gene diversification mainly include techniques such as error-prone polymerase chain reaction (epPCR; a shotgun technique), saturation mutagenesis (SM; focused randomization), and DNA shuffling (a recombinant technique). Currently, since the bottleneck remains to be the throughput of screening/selection, the rational enzyme designing methodologies emerged. “Smart” libraries can in principle offer higher hit rates of improved variants.

From the above starting point, both directed evolution and rational enzyme design have received enormous research interests and the advances surged in the relevant fields. In the following sections we will demonstrate how the state-of-art strategies were employed in the applications for improve enzyme stability and benefit the real life.

**Fig. 1:**
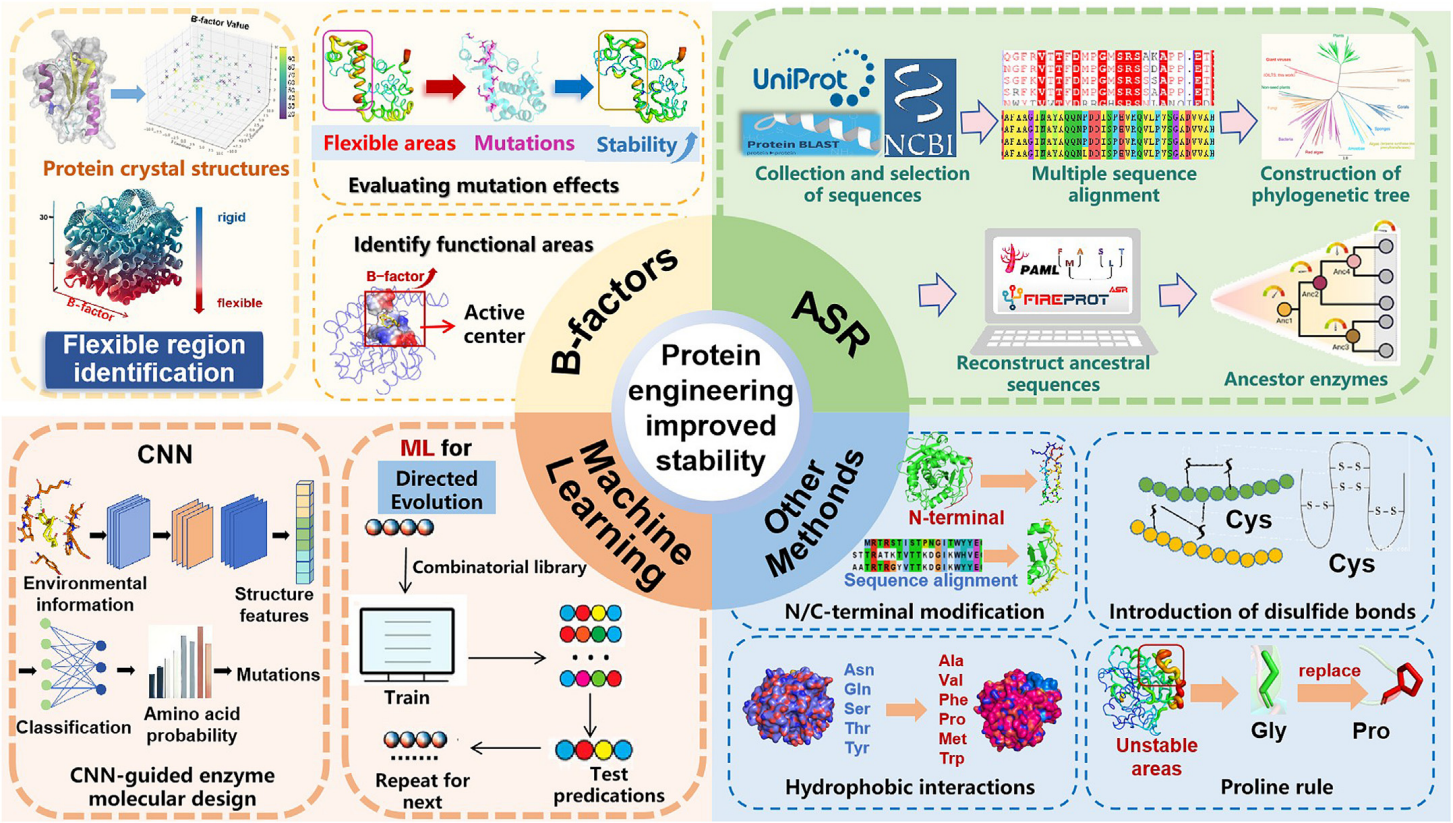
Protein engineering strategies for enhancing enzyme stability.

In organic chemistry and biotechnology, operation conditions often involve organic solvents, long-term reactions, and relatively high temperatures. One of the weaknesses of enzymes compared to organic or metal catalysts is the resistance to harsh conditions. Therefore, to evolve enzymes to accommodate industrial operating conditions has become one of the major goals for protein engineers. As shown in Fig. 1, traditional strategies for enhancing enzyme stability include the introduction or engineering of disulfide bonds, salt bridges, hydrogen bond networks, hydrophobic cores, and other structural modifications. B-factor is routinely employed to identify key residues influencing stability, evaluate overall protein stability, and pinpoint functional regions [15]. In recent years, ancestral sequence reconstruction (ASR) has gained prominence for its applications in discovering novel enzymes and guiding stability-enhancing mutations [16]. Similarly, machine learning (ML) has begun to emerge as a powerful tool for protein engineering, offering data-driven insights to facilitate protein design and modification [17].

### 1.2 Protein engineering for improved thermostability using B-factors

The B-factor, also referred to as the Debye-Waller factor or temperature factor, is a critical parameter used to describe the intrinsic thermal motion of atoms in a protein crystal lattice [18]. X-ray structures available in the Protein Data Bank (PDB) provide B-factors for all atoms, including hydrogen, though B-factors for hydrogen atoms are typically not explicitly listed. In the absence of X-ray structures, computational tools have been developed to predict B-factors, broadening their utility [[19], [20], [21], [22]].

**Fig. 2:**
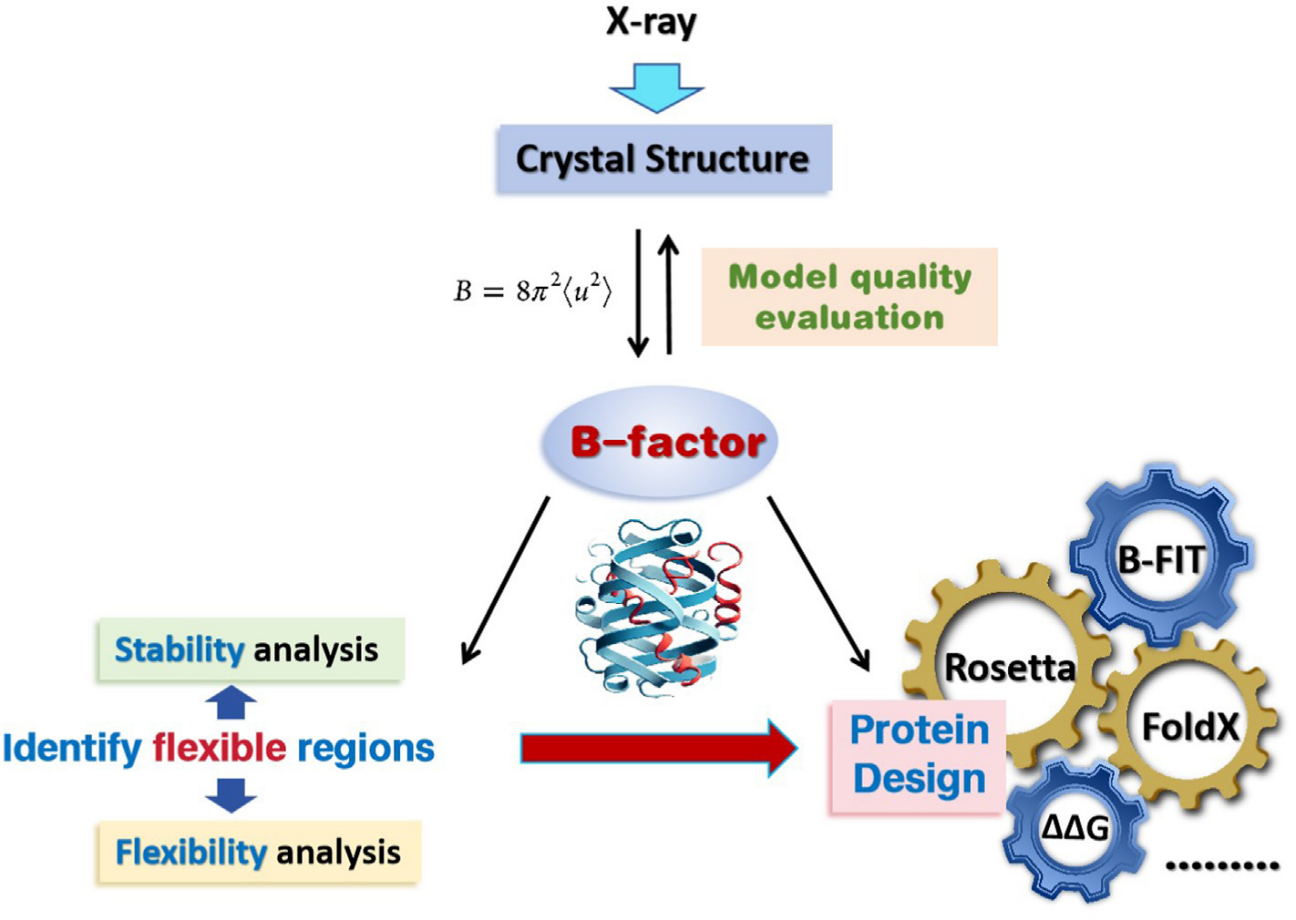
B-factor identification of protein flexible regions and its application in stability design.

As illustrated in Fig. 2, abnormal B-factor distributions in X-ray crystallography may indicate errors in structural models or biases in experimental data, making B-factors a valuable metric for evaluating model quality. Since B-factors quantify atomic positional uncertainty, they are closely associated with protein thermal stability. Regions with elevated B-factors typically exhibit reduced stability and are thus prime targets for mutagenesis aimed at enhancing thermal stability. Common mutagenesis approaches include B-factor iterative testing (B-FIT) [15] and computational tools such as FoldX [23] and Rosetta [24]. Strategic mutations in these regions often lead to significant improvements in thermal stability.

In a recent study, Cabanding et al. [25] employed B-factor analysis to evaluate the impact of different helical mutations on the structural stability of lipase (PAL). Proline residues, due to their side-chain-induced steric hindrance, can disrupt the integrity of α-helices, leading to structural alterations. By designing proline mutations to intentionally destabilize α-helices, the researchers monitored the B-factors of each residue in both native and mutant proteins to identify regions most affected by structural perturbations. Furthermore, B-factors were used to quantify structural deviations and to track changes in cavity radius, enabling the assessment of how these mutations influenced catalytic behavior. Among the mutants tested—A34P, A115P, R119P, and L159P—the R119P variant exhibited a remarkable increase in activity, achieving a nearly ninefold enhancement during a 180 ​min reaction. B-factor analysis revealed that the R119P mutant features a wider gorge radius and greater structural flexibility in its open conformation, likely contributing to its improved enzymatic activity.

The B-factor is widely utilized to identify flexible regions within proteins; however, it does not provide specific guidance for mutating these flexible sites. Employing a saturation mutagenesis strategy often leads to substantial increases in experimental costs and time requirements. To address this, Ru et al. combined B-factor analysis with the Rosetta [24] to facilitate virtual mutagenesis, utilizing ΔΔG values to guide the construction of mutant libraries and thereby avoiding the direct experimental burdens of saturation mutagenesis. A negative ΔΔG value is commonly recognized as an indicator that a point mutation can enhance protein stability, with smaller ΔΔG values generally correlating with increased stability [26]. In this study, the flexible regions of dextranase SmdexTM were identified based on its crystal structure, with the ten amino acid sites exhibiting the highest B-factors selected for analysis. Subsequently, Rosetta was employed for virtual saturation mutagenesis, yielding 12 single-point mutants with the lowest ΔΔG values for experimental validation. The results demonstrated the successful identification of four single-point mutants and three double mutants, all of which exhibited significant improvements in thermal stability. Notably, the combined mutations N102P/D500G and N102P/D500T resulted in a 5 ​°C increase in the optimal temperature, with half-lives at 45 ​°C extending by 3.14-fold and 2.44-fold, respectively [27].

Enzyme active sites, in contrast, generally exhibit lower B-factors, indicating reduced flexibility, a feature essential for catalytic efficiency. This makes B-factors not only valuable for identifying flexible, unstable regions but also for highlighting catalytic centers. By focusing on flexible regions, whether they are specific amino acids or larger structural motifs, targeted mutations can improve protein stability [15].

**Fig. 3:**
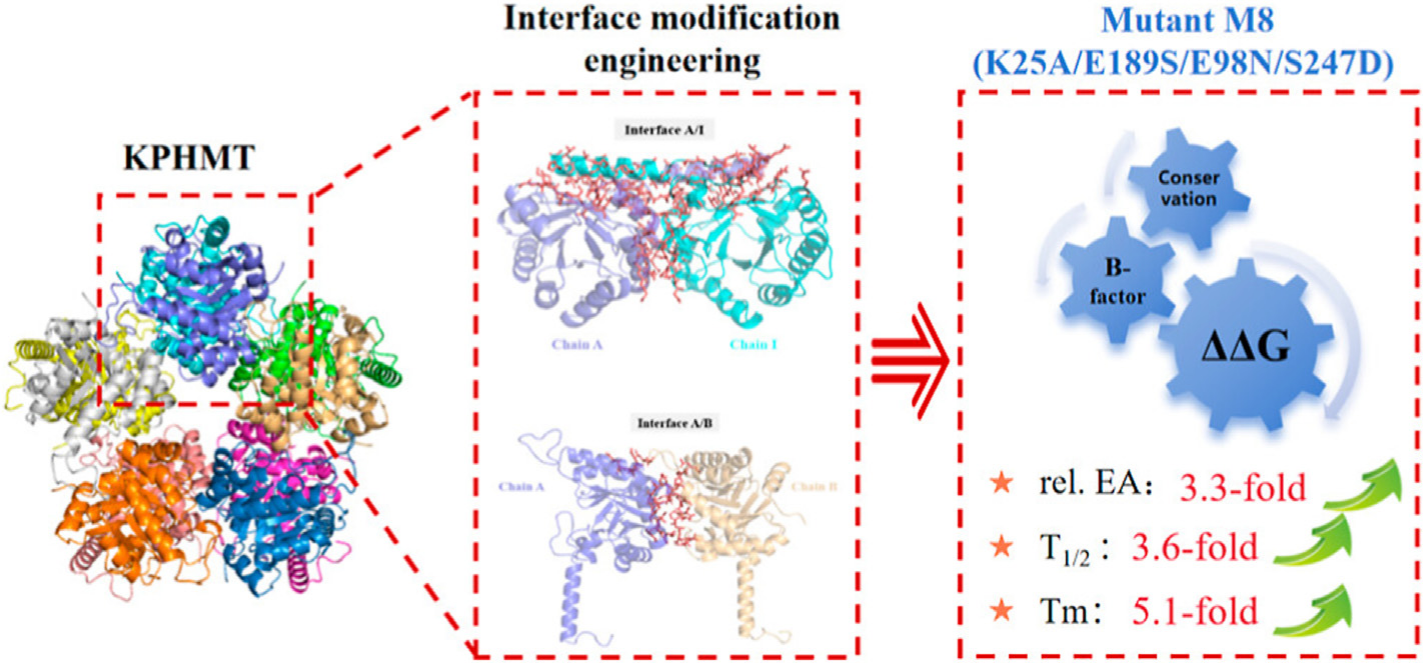
Design of KPHMT by ΔΔG of the interface and B-factor analysis [29]. Copyright 2024, American Chemical Society.

Hong et al. performed a comprehensive analysis of thermophilic and mesophilic proteins, using B-factors, molecular dynamics, and structural features to demonstrate that surface cavities or boundary regions significantly influence protein stability [28]. For multi-subunit enzymes, the stability of inter-subunit interfaces plays a key role in overall protein integrity, as dissociation often begins at these interfaces. In a study by Xue et al. [29], folding free energy calculations (ΔΔG), B-factor analysis, and B-FITTER were utilized to identify candidate mutation sites at the dimeric interface of ketopantoate hydroxymethyltransferase (KPHMT). By using the DeepDDG [30] web tool to estimate the effects of mutations on ΔΔG, and applying B-FITTER to predict flexible residues, mutations with excessively high B-factors were screened. The remaining 25 candidates were subjected to single-point mutagenesis, with three key sites chosen for further saturation and combinatorial mutagenesis. As illustrated in Fig. 3, under the guidance of this combination of various strategies, the optimal double mutant M8 was achieved, which increased the enzyme's half-life at 30 ​°C from 8.83 to 15.44 h—equivalent to a 3.29-fold improvement. These findings illustrate the utility of B-factors in guiding protein engineering to improve stability.

In another study, machine learning models were combined with B-factor data to identify mutational hotspots in the endoglucanases EGI and EGII. By prioritizing flexible regions for mutation, Gao et al. developed four thermostable variants, achieving a dramatic increase in residual activity at 60°C—from 40 ​% to over 90%—alongside significant improvements in catalytic performance [31].

Despite these successes, enhancing thermostability through mutagenesis often comes with trade-offs, particularly with catalytic efficiency. For example, in research on protease PB92, a scoring system was implemented to balance these competing factors. B-factor analysis revealed several residues with high values, leading to the generation of 21 single-point mutants, of which 15 exhibited increased thermal stability. The G100E variant extended the enzyme's half-life at 65 ​°C by a remarkable 23.7-fold. However, reductions in activity were noted in some mutants. To mitigate this, a scoring system was introduced that considered both activity and stability changes, resulting in beneficial mutants including N18L/R143L/S97A and N18L/R143L/G100A. These variants demonstrated significantly improved thermostability and catalytic efficiency metrics three times higher than those of the wild-type enzyme, highlighting their industrial potential [32].

**Fig. 4:**
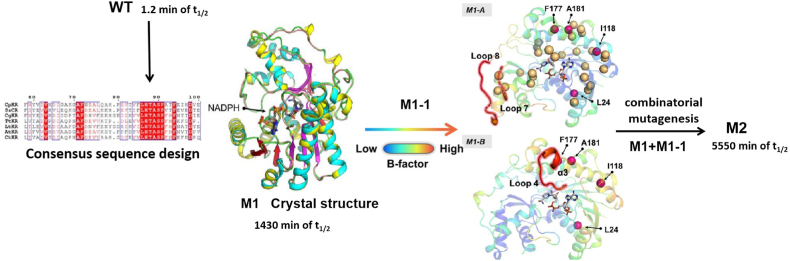
Combined mutagenesis of rigid and flexible regions accomplished by consensus sequence design and B-factor analysis [33]. Copyright 2023, American Chemical Society.

Chen et al. employed a dual strategy targeting both rigid and flexible regions of ketoreductase CpKR to enhance thermostability, as illustrated in Fig. 4 [33]. By focusing on residues with high B-factors and identifying key residues in rigid regions, they generated the optimal mutant M1, which extended the enzyme's half-life at 40 ​°C from 1.2 ​min to 1430 ​min. Structural analysis revealed 40 high B-factor residues and 12 regions of missing electron density, providing the foundation for a mutagenesis library of 1000 variants. Screening with tools such as FoldX [23] and Rosetta Cartesian ddg [34] identified 87 beneficial mutations, with 10 showing enhanced thermostability. The best mutations were combined to create the quadruple mutant M2, which further increased the half-life at 40 ​°C to 5550 ​min—a 463-fold improvement over the wild-type enzyme.

While B-factors serve as a valuable indicator for identifying unstable regions within proteins, their reliability can decrease at lower resolutions. At reduced resolution, B-factors may be artificially elevated, and their correlation with root mean square deviation (RMSD) may weaken due to factors like lattice disorder. For proteins lacking crystallographic data, computational tools such as PROFbval [20], MoRFpred [35], and ResQh [36] have been developed to predict B-factors and guide mutagenesis strategies [15]. For instance, in the studieson D-allulose 3-epimerase (RbDAEase), structural alignments from homologous proteins in the PDB database were used to predict B-factors, and mutations at these sites successfully improved thermal stability [37]. While computational methods have greatly expanded the toolkit for enhancing protein thermostability, they still fall short of fully replicating the detailed insights provided by experimental B-factor data, especially when it comes to capturing the subtle aspects of protein flexibility.

In summary, the B-factor, derived from X-ray crystallography, has proven indispensable in identifying regions of thermal instability within proteins. Its application, particularly when integrated with site-directed mutagenesis and advanced computational tools, has led to significant advancements in optimizing both thermostability and enzymatic activity. As machine learning and computational methods continue to evolve, the utility of B-factors in protein engineering will likely expand, further advancing the field by offering deeper insights into protein flexibility and stability.

### 1.3 Advancing thermostable protein engineering through ancestral sequence reconstruction

Ancestral sequence reconstruction (ASR) is a computational approach employed to infer the amino acid sequences of ancestral enzymes from extinct species [38]. Initially introduced by Stackhouse et al., in 1990, ASR was primarily used to test evolutionary hypotheses and study molecular evolution [39]. A pivotal finding from these early investigations was the consistently higher thermostability of resurrected ancestral enzymes, often exhibiting denaturation temperatures far surpassing those of contemporary enzymes [40]. This pronounced thermal resilience suggests that early proteins were adapted to the extreme environmental conditions of ancient Earth, likely retaining thermophilic characteristics [41]. In addition to their stability, ancestral enzymes frequently exhibit superior catalytic activity and expression levels, establishing ASR as a valuable technique not only for evolutionary studies but also for the rational design of robust industrial enzymes with enhanced functional properties [42,43].

The reconstruction of ancestral enzymes involves several critical stages, including sequence collection and selection, multiple sequence alignment, phylogenetic tree construction, and the inference of ancestral sequences [16]. The selection of a diverse and representative sequence set, alongside accurate phylogenetic tree construction, is essential for ensuring the precision and reliability of the reconstructed ancestral enzymes. Over time, ASR algorithms have undergone substantial advancements, with Maximum Likelihood (ML) and Bayesian inference emerging as the dominant methods for ancestral sequence inference. Widely adopted tools like PAML and MrBayes are integral to implementing these approaches effectively [44,45].

To meet the increasing demand for accessible ASR methodologies, integrated platforms such as FireProtASR [46], FastML [47], and PhyloBot [48] have been developed, streamlining the reconstruction process. These platforms consolidate key steps—including multiple sequence alignment, phylogenetic tree construction, and ancestral sequence inference—into a unified, user-friendly interface. Requiring only minimal input, typically in the form of protein sequences in FASTA format, these tools significantly lower the technical and computational barriers that traditionally constrained ASR. Consequently, they have broadened the accessibility of ASR to a wider range of researchers, enhancing its applicability in fields like protein engineering and enzyme design.

**Fig. 5:**
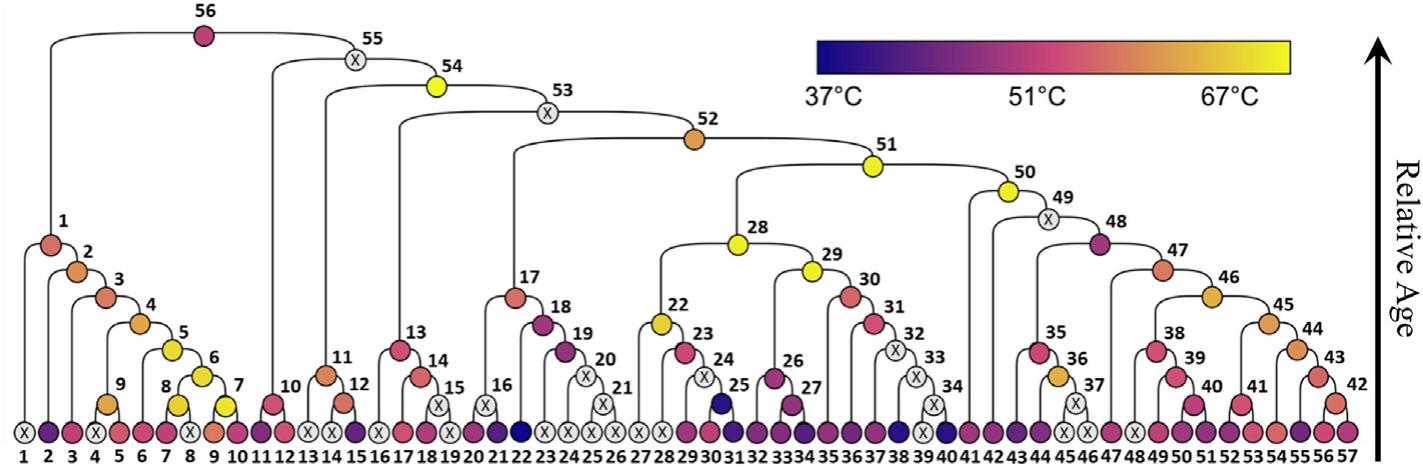
Phylogenetic tree of the EREDs [49]. Copyright 2023, American Chemical Society.

For example, Lewis et al. utilized FireProtASR [46] to generate a large panel of 113 ene reductases (EREDs), comprising 56 homologous sequences of the template enzyme EBP1 and 56 ancestral sequences, as illustrated in Fig. 5. These sequences shared between 45 ​% and 95 ​% identity, and 101 out of the 113 enzymes were successfully expressed in *E. coli*. The ancestral enzymes, on average, exhibited thermostability improvements of about 9 ​°C compared to their modern counterparts, although the extent of thermostabilization was not directly correlated with the age of the ancestral enzymes. Furthermore, no significant correlations were found between the activity levels of wild-type and ancestral enzymes. However, a notable reduction in enantioselectivity was observed, supporting the hypothesis that ancestral enzymes tend to have broader substrate specificities, which may trade off with enantioselectivity [49].

In another successful application of ASR, Ni et al. enhanced the thermostability of an alcohol dehydrogenase (KpADH). From a phylogenetic analysis, two modern ADHs (D10 and D11) were identified, and three ancestral sequences were reconstructed, two of which (A61 and A64) displayed moderate activity and stereoselectivity toward a model substrate (1.5 and 4.3 U ​mg^−1^, respectively). D10 and A64 were then thoroughly characterized for their pH profiles, temperature stability, substrate scope, and catalytic kinetics. The ancestral enzyme A64, in particular, exhibited a broader substrate scope and significantly improved thermostability compared to the modern KpADH enzyme, demonstrating the potential of ASR to uncover superior biocatalysts for industrial applications [50].

It is well-recognized that there is often a trade-off between activity and stability in enzyme engineering, a challenge exemplified by the CO_2_-fixing enzyme Rubisco [51]. Notably, the reconstruction of ancestral Rubisco uncovered an accessory subunit that likely played a role in stabilizing the enzyme before the advent of atmospheric oxygen 2.4 billion years ago [52]. Other software aids including SCHEMA [53], Constrained Network Analysis [54] and FRESCO have also been applied in the identification of flexible regions of proteins and improve the thermostability.

**Fig. 6:**
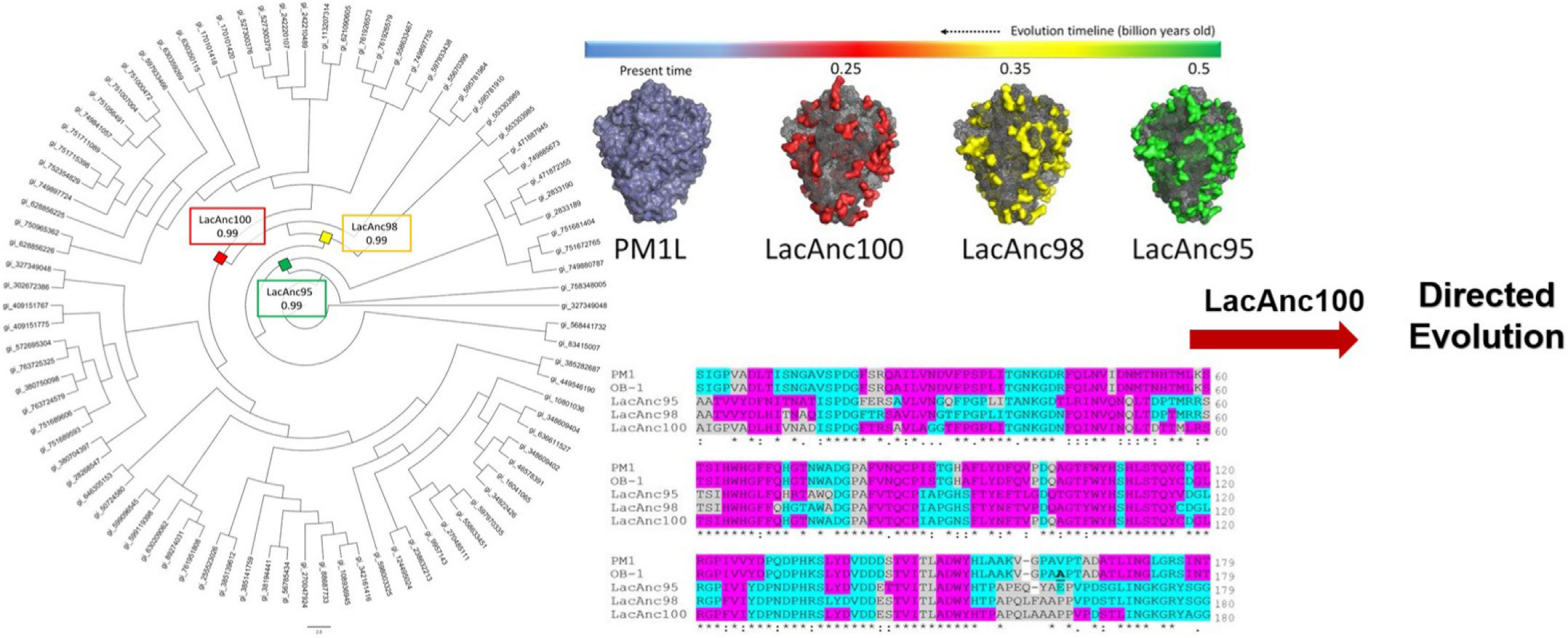
Ancestral sequences as the starting point for directed evolution [55]. Copyright 2020, American Society for Microbiology. Published under a CC-BY license.

One of the most compelling advantages of ancestral enzymes, as shown in Fig. 6, is their increased thermostability, which confers greater evolutionary plasticity relative to modern enzymes, making them promising candidates for protein engineering [42]. A notable example of this potential was demonstrated by Gomez-Fernandez et al. [55], who successfully reconstructed three ancestral laccases using PAML. Among the reconstructed variants, LacAnc98 and LacAnc100 exhibited enhanced stability and improved secretion compared to their modern counterparts. Using LacAnc100 as a template for directed evolution, random mutagenesis and subsequent screening led to the identification of a mutant with a 160 ​% increase in the oxidation rate of the substrate 3-cyclopentene-1,2-dione.

**Fig. 7:**
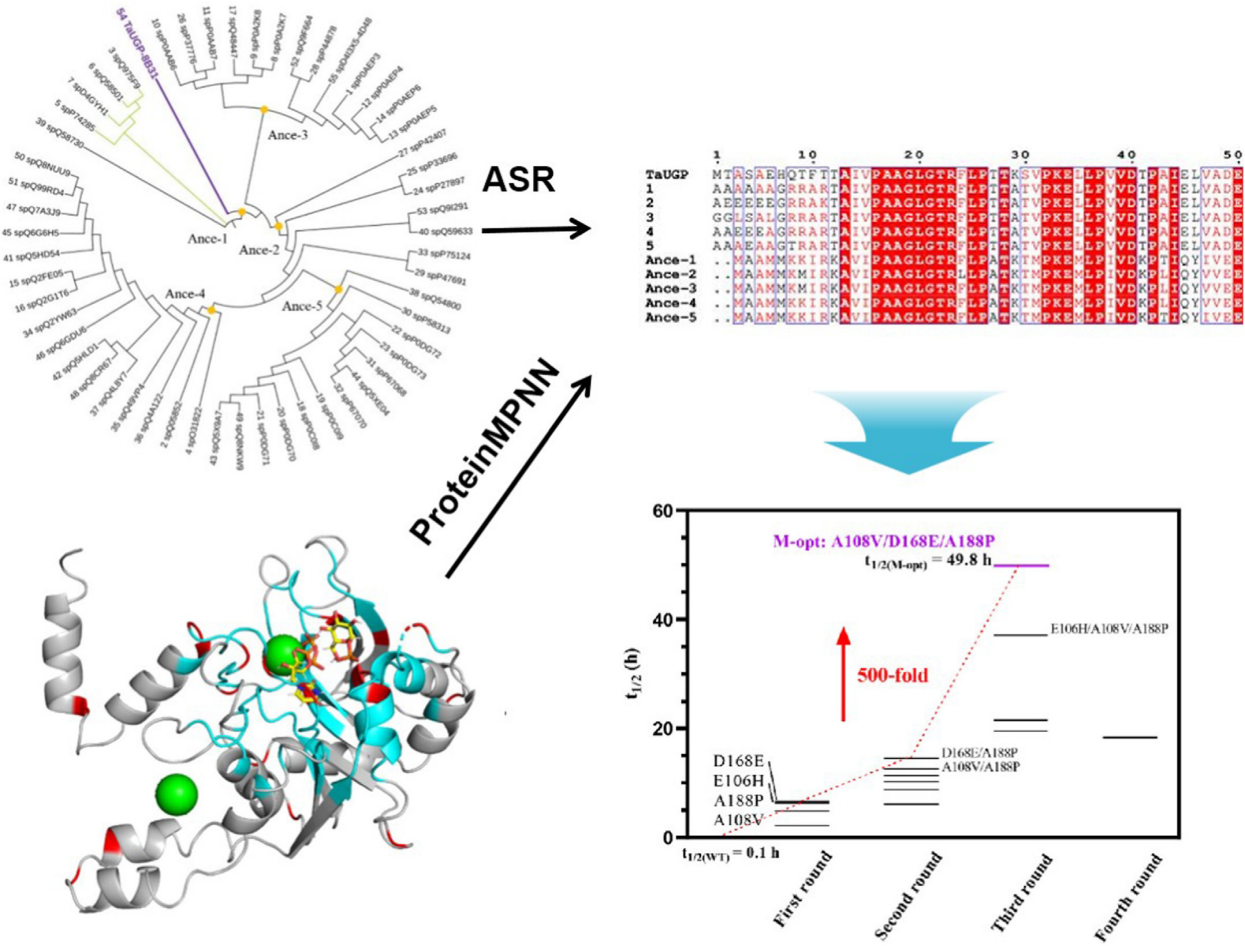
Deep learning and ASR combined to identify mutation sites using multiple sequence comparison [56]. Copyright 2024, American Chemical Society.

Building on this success, another study, as illustrated in Fig. 7, exemplified the integration of ancestral sequence reconstruction (ASR) with deep learning techniques in the design of UDP-glucose pyrophosphorylase (UGP) [56]. To maintain the integrity of the substrate-binding pocket, the researchers constrained the amino acids within a 12 ​Å radius to prevent any disruption of catalytic activity. Employing the deep learning model ProteinMPNN, they identified critical residues, which led to the generation of 10 initial sequence variants. These variants were subsequently integrated with ancestral sequences reconstructed using PAML from 55 homologs obtained from UniProt, ultimately resulting in the selection of five ancestral nodes for further investigation. After conducting a multiple sequence alignment of these ancestral and modern variants, 16 candidate mutations were tested. Four single-point mutations successfully led to improved thermal stability, and further screening of the recombed mutants yielded the optimal triple mutant A108V/D168E/A188P. This variant exhibited a remarkable 500-fold increase in half-life at 60 ​°C while retaining 90 ​% of the wild-type activity, demonstrating the power of ASR when integrating with cutting-edge machine learning techniques. These cases underscore the potential of ASR not only as a tool for understanding evolutionary adaptations, but also as a powerful starting point for rational enzyme design.

As shown in Table 1, currently, ASR is mostly regarded as a tool for the discovery of new enzymes in enzyme engineering. Despite the encouraging progress, the applications of ASR in protein discovery and design remain limited. One major constraint is the inherent uncertainty associated with the technique. While computational methods can infer the most likely ancestral sequences, there is no definitive way to confirm that the reconstructed sequences precisely replicate that existed in the past. When ASR is applied as a tool for enzyme mining, it is difficult to control the properties of the reconstructed ancestral enzymes; for example, improvements in thermal stability may be accompanied by a decrease in activity. Another challenge is the level of computational expertise historically required for ASR, which could be a barrier for experimentalists. However, the development of user-friendly, one-step ASR platforms has substantially simplified the process, reducing the technical threshold for its application. Additionally, advancements in gene synthesis technologies, coupled with the decreasing costs of synthetic sequences, are expected to further propel the widespread adoption of ASR for discovering thermostable and highly active novel enzymes, positioning the technique as a transformative tool in the field of enzyme engineering.

**Table 1:** Examples of ancestral enzyme applications in the article and in recent years.

Enzyme	ASR protocol	Usage of ASR	References
Ene reductases	FireProtASR	Enzyme mining	46
Alcohol dehydrogenase	FASTML	Enzyme mining	50
Laccases	PAML	Consensus sequence alignment	55
UDP-glucose	PAML	Consensus sequence alignment	56
P450 monooxygenases	GRASP	Enzyme mining	57
Phenolic acid decarboxylase	GRASP	Enzyme mining	58
PETase	GRASP, PAML	Enzyme mining	59

### 1.4 Protein engineering for improved thermostability by machine learning

With the ongoing advancements in computational power, protein databases, and sequencing technologies, machine learning (ML)-guided protein engineering is emerging as an important tool for enzyme design and modification [60]. ML functions by inputting specific datasets and detecting inherent patterns to predict the properties of previously uncharacterized entities with similar attributes. The input data, known as the training set, is fundamental to the success of ML. It extracts relevant features from these datasets, which include enzyme molecular sequences, spatial structures, and physicochemical properties, allowing researchers to model and predict enzyme behavior more efficiently [61,62].

Currently, publicly available protein databases such as NCBI [63], PDB [65], and UniProt have amassed vast amounts of data. These databases contain thousands of protein structures, millions of sequence entries, thousands of biophysical measurements, and numerous annotations of catalytic mechanisms. This ever-expanding repository of information enables scientists to extract and use this data to train ML models, significantly advancing the field of protein structure prediction and design. For example, deep learning approaches have revolutionized protein structure prediction, with tools such as AlphaFold2 [66], developed by Demis Hassabis and John M. Jumper, and RoseTTAFold [67], created by David Baker, achieving high-precision predictions of protein structures directly from amino acid sequences. These breakthroughs circumvent the high costs and difficulties associated with experimental protein crystallization, establishing a solid foundation for rational or semi-rational protein design. David Baker's Rosetta software, in particular, facilitates *de novo* protein design by defining protein backbones based on functional requirements [68]. In recognition of their contributions, Hassabis, Jumper, and Baker were collectively awarded the 2024 Nobel Prize in Chemistry.

In addition to structural analysis, ML methods have facilitated the development of various tools for predicting key protein properties. These tools span a range of applications, such as classifying enzymes [69], predicting protein thermostability through convolutional neural networks (CNNs) [70], and assessing catalytic activity in the GT1 family of glycosyltransferases [71]. Other notable examples include the prediction of enzyme solubility, catalytic residues using random forest-based models such as PREvaIL [72], and the identification of active sites via 3D convolutional neural networks [73]. To bridge the gap between the flourishing ML-based enzyme property predictions and the challenges in direct enzyme engineering applications, it is essential to recognize the critical role that data availability plays in both areas. While tools for predicting enzyme properties are developed and increasingly accurate, applying ML directly to enzyme engineering remains underexplored. This limitation is largely due to the scarcity of sufficiently large, publicly available datasets that are necessary to train robust models. Experimental datasets, whether derived from literature or through rational design efforts, often lack the volume and diversity required to support reliable training and validation of ML models [74]. Therefore, constructing more comprehensive, balanced, and high-quality databases will be crucial for advancing ML in the future.

**Fig. 8:**
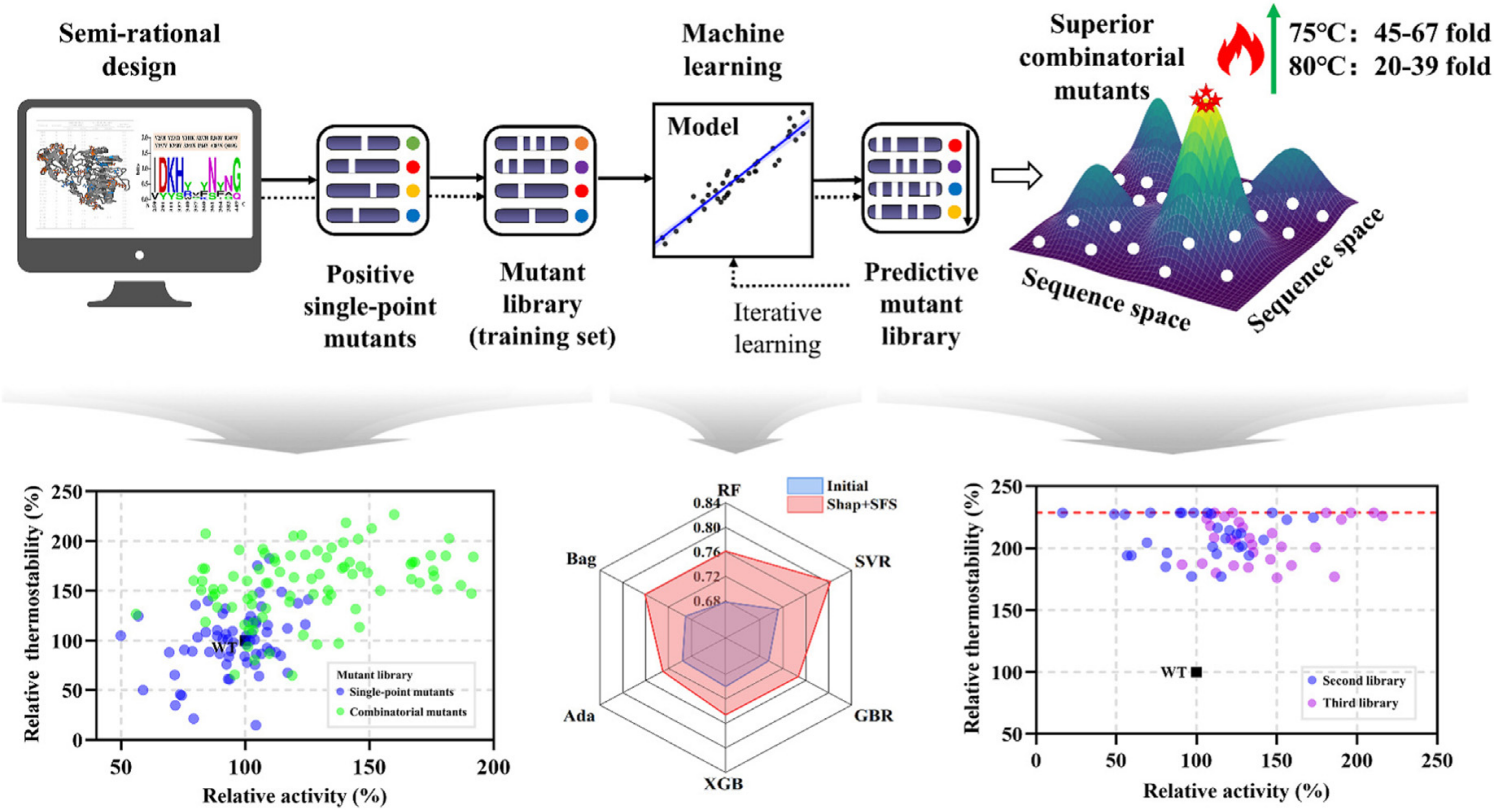
Using ML to predict the optimal combinatorial mutant at multiple mutation sites [73]. Copyright 2024, Elsevier. Published under a CC-BY license.

As illustrated in Fig. 8, the study by Zhang et al. addressed the challenge of predicting multi-site combinatorial mutations in pectate lyase (PNL) with limited data samples, successfully enhancing the enzyme's thermostability [73]. Initially, they identified 54 potential mutation sites using FireProt [36] and BayeStab [74], along with 12 additional sites identified through consensus sequence alignment. After an initial screening, 18 single-point mutants were identified with enhanced thermostability. Subsequently, a random combinatorial mutagenesis approach was employed, resulting in a library of 106 mutants, including 88 combinatorial variants. The researchers also incorporated 46 non-beneficial single-point mutants identified earlier into the dataset to build an ML model. The model used enzyme sequences as input and predicted relative residual activity as output. Given the dataset's limited size, 10-fold cross-validation was applied to assess performance, and further refinement was achieved using SHAP algorithms and sequential forward search (SFS) to enhance predictive accuracy. Through these techniques, two combinatorial mutants, P36 and P48, were identified with remarkable improvements in thermostability, extending half-lives at 75 ​°C by 67-fold and 61-fold, respectively. This study underscores the power of ML in guiding multi-site combinatorial mutagenesis, demonstrating significant potential in improving enzyme properties through data-driven approaches.

Random mutagenesis experiments generate substantial data; however, many resulting mutants may be functionally irrelevant or detrimental. Although these unproductive mutations are disregarded, but such data hold substantial value for ML-guided enzyme engineering [75]. The primary cost associated with exploiting these datasets is DNA sequencing, which is becoming increasingly affordable. Even when the underlying biophysical mechanisms are not fully understood, ML models can still generate valuable predictions from the available data.

However, sequencing every variant and synthesizing selected mutations could impose additional financial and experimental burdens. When screening costs are high, or when the process is too slow to justify sequencing and synthesis, ML proves especially useful. In cases where gene synthesis is required for mutant libraries—rather than traditional mutagenesis—ML can strategically select sequences to synthesize, optimizing the efficiency of the experimental workflow.

**Fig. 9:**
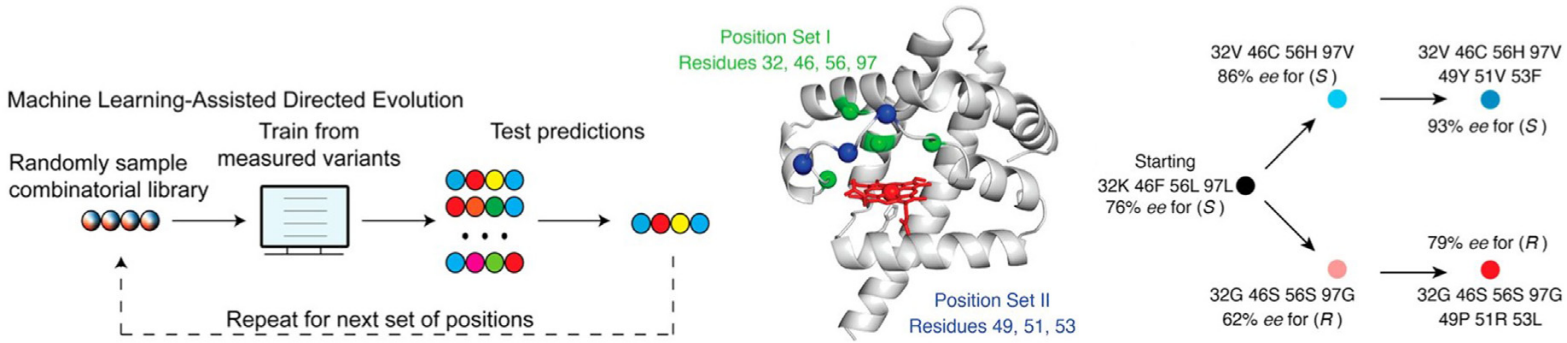
ML-guided directed evolution achieves enantioselectivity of NOD enzyme [76]. Copyright 2019, Proceedings of the National Academy of Sciences.

One example of this is the study by Wu et al. [76], where the researchers initially validated their approach by referencing the investigation of GB1 protein-antibody interactions conducted by a different group of Wu et al. [77], as illustrated in Fig. 9. They compared the final fitness outcomes of simulated directed evolution with and without the aid of ML, confirming the ability of ML to guide and accelerate evolutionary processes. Following this validation, they applied the method to directed evolution of nitric oxide dioxygenase (NOD), demonstrating its effectiveness in facilitating enzyme optimization. After each evolutionary round, the ML model was updated with new data, enhancing its predictions for subsequent mutations. Unlike traditional methods, the ML-driven approach enabled exploration across a broader sequence space, accelerating the identification of optimal variants. The results demonstrated that the ML-guided method significantly enhanced the efficiency of predicting variant fitness, even when the accuracy of the models was imperfect. Subsequently, the researchers employed the same ML framework to evolve nitric oxide dioxygenase (NOD) for the enantioselective production of both (S)- and (R)-enantiomers. Through two rounds of directed evolution, the ML approach optimized the mutant libraries, ultimately identifying enzyme variants with substantially improved enantioselectivity. Specifically, the most selective variant achieved an enantioselectivity of 93 ​% for the (S)-enantiomer, while a variant for the (R)-enantiomer reached 79 ​% enantioselectivity. This work underscores the promise of ML-assisted directed evolution, highlighting its capacity to accelerate the optimization process and enhance biocatalyst performance. The findings indicate that ML can facilitate significant advancements in enzyme engineering, enabling notable improvements in catalytic activity and selectivity without necessitating a complete understanding of the underlying biophysical mechanisms.

Deep learning, a subset of ML, relies on neural networks and typically requires large-scale datasets to achieve optimal performance [78]. This methodology effectively captures the diversity inherent in natural protein sequences, thereby facilitating the generation of protein variants that broaden the functional protein space [79]. As previously mentioned, models such as AlphaFold [64] and Fang et al.'s [68] thermostability predictor have successfully employed CNNs to make major strides in protein structure and stability prediction. CNNs, with their ability to capture spatial hierarchies, are particularly well-suited for analyzing protein structures, where local and global residue interactions are critical to function and stability [80,81]. For example, Lu et al. employed a self-supervised CNN named MutCompute to identify stabilizing mutations in enzymes [82]. This algorithm was trained on localized chemical microenvironments of amino acids from over 19,000 proteins in the PDB database. The researchers constructed microenvironments around enzyme active sites to analyze the relationship between residues and their surroundings. The data were voxelized into seven distinct channels, which were then input into the MutCompute model. Through convolutional and max-pooling layers, the model extracted microenvironmental features, which were used to classify the likelihood of each amino acid being the center residue in its microenvironment. This approach enabled precise targeting of residues for mutagenesis. Using these insights, the team optimized FAST-PETase, an enzyme that degraded 33.8 ​mmol of terephthalic acid monomers within 96 ​h at 50 ​°C, demonstrating significant improvement in stability and performance.

Table 2 incorporates exemplary instances culled from the text, in tandem with recent applications of ML within the ambit of enzyme engineering. The rapid evolution of AI, particularly ML, has revealed tremendous potential in enzyme engineering, despite some challenges. A key obstacle is the limited availability of comprehensive datasets covering essential enzyme properties like reaction mechanisms, substrate specificity, and cofactor interactions. Additionally, the lack of sufficient negative data—mutations leading to poor performance—reduces the effectiveness of ML in predicting unfavorable mutations. Nevertheless, the future of ML in enzyme engineering is promising. As high-quality experimental data continues to accumulate and ML algorithms evolve, the focus is increasingly shifting toward more complex tasks, such as optimizing multi-site mutations, improving enzyme stability, and predicting novel catalytic activities. As these advancements continue, ML is poised to become an indispensable tool for enzyme design, enabling faster, more precise improvements in enzyme performance while minimizing the need for extensive trial-and-error experimentation.

**Table 2:** Examples of machine learning applications to enzyme engineering in the article and in recent years.

Enzyme	Algorithms	Input types	References
Pectate lyase	Multiple integrated learning algorithms	Sequences	73
Nitric oxide dioxygenase	Shallow neural networks	Sequences	76
PET hydrolases	CNN	Structures	82
Lysine decarboxylase	MutComputeX	Structures	83
Acetylcholinesterase	RF	Molecules	84
N-acetyl-L-glutamate kinase	PREVENT	Sequences	85
Vitreoscilla hemoglobin	Polynomial Naive Bayes Classifier	Sequences	86
Carboxylesterases	GBRT	Sequences and biochemical properties	87
Amidase	RF	Substrate structure characterization	88

CNN: Convolutional neural network; RF: Random forest; PREVENT: Predictive Enzyme Variant; GBRT: Gradient Boosting Regression Tree.

### 1.5 Protein engineering for enhanced resistance against organic solvents

Organic solvents could negatively impact the catalytic activity of the enzymes in a number of aspects such as causing conformational changes, loss of essential water molecules of the proteins, or competitive inhibition, etc. Since in most industrial productions, solvents are inevitable for substrate solubilization, product extraction, or other process requirements, efforts were invested in protein engineering to improve the resistance to solvents. Reetz et al. demonstrated that for the lipase from *Bacillus subtilis* (BSL), the mutants with enhanced thermostability derived from B-FIT/ISM studies, also displayed high resistance towards polar organic solvents including acetonitrile (ACN), dimethylsulfoxide (DMSO) and dimethylformamide (DMF) [89]. Since then, correlations between good thermo- and solvent stability have been established in general, as demonstrated by a number of investigations. Salt bridges were targeted in a study by Schwaneberg et al., who improved both the thermostability (up to 137-fold) and resistance to solvents (up to 7.6-fold) of the enzyme *Bacillus subtilits* Lipase A (BSLA). The landscape of the salt bridges in the saturation mutagenesis libraries of BSLA were analyzed, and a few ‘unfavorable salt bridges’ were identified from the molecular dynamics (MD) studies [90]. Theses unfavorable salt bridges were broken by substitutions with oppositely charged residues that may restore the surface interaction network, thereby stabilizing the proteins. In the end the substitutions have effectively collapsed the salt bridges and also increased the flexibility at local regions in organic solvents and high temperatures.

In enhancing solvent resistance, CompassR [91], a computational tool, predicts solvent-binding hotspots, enabling targeted mutations at these sites to improve enzyme solvent tolerance. Additionally, surface polarity and charge engineering have gained significant attention in recent years for their potential in this area. For example, Cui et al. [92] enhanced the organic solvent resistance of *Bacillus subtilis* lipase A (BSLA) by introducing polar substitutions at surface-exposed amino acids. A total of 45 BSLA variants were selected, including 30 beneficial polar substitutions and 15 detrimental ones, with mutation sites covering 15 surface-exposed positions across the enzyme. Molecular dynamics (MD) simulations revealed that most beneficial variants exhibited a reduction in the number of local DMSO molecules and an increase in the number of water molecules. Additionally, the root-mean-square fluctuation (RMSF) values of the beneficial variants were significantly lower than those of the detrimental variants, indicating that the polar substitutions may enhance enzyme stability in DMSO by altering the distribution and orientation of solvents. The analysis of experimental data for 1631 polar-related variants further revealed that polar substitutions most notably improved resistance to DMSO, with mutations replacing aromatic amino acids with polar ones contributing to increased enzyme stability while maintaining a low mortality rate.

In the research conducted by Sorgenfrei et al. [93], a systematic evaluation of a representative set of ene-reductases (EREDs) led to the introduction of a novel parameter, C*T U50*, designed to better characterize enzyme behavior across varying co-solvent concentrations. The findings indicate that while the melting temperature (Tm) is commonly utilized as an indicator of thermal stability, its correlation with enzymatic activity is not straightforward; conversely, C*T U50* effectively reflects the balance between enzyme folding and unfolding, demonstrating a significant association with enzymatic activity. Analyzing thirteen EREDs, the study employed an activity model incorporating Gaussian terms. Although all enzymes exhibited thermal instability in the presence of co-solvents, their catalytic activities varied with co-solvent concentrations, with lower concentrations even enhancing the activity of certain enzymes. This observation prompted the definition of C*T U50* as the co-solvent concentration at which half of the enzyme molecules are folded or unfolded at a specific temperature, establishing a significant relationship between C*T U50* and the concentration at which activity loss occurs. The research offers a fresh perspective on assessing enzyme stability and catalytic performance in organic solvents, paving the way for further exploration of enzyme behavior in complex environments.

Thermostability and solvent stability were simultaneously enhanced when an alcohol dehydrogenase from *Pseudomonas putida* KT2440 (PedE) was engineered. A small and focused library of only 200 clones were constructed and the best mutations were combined in the subsequent rounds. The best-performing triple mutant exhibit a 7 ​°C increase in thermostability and 2-fold increase in residual activity towards incubation for 1 ​h in 50 ​% of dimethyl sulfoxide (DMSO) [94].

**Fig. 10:**
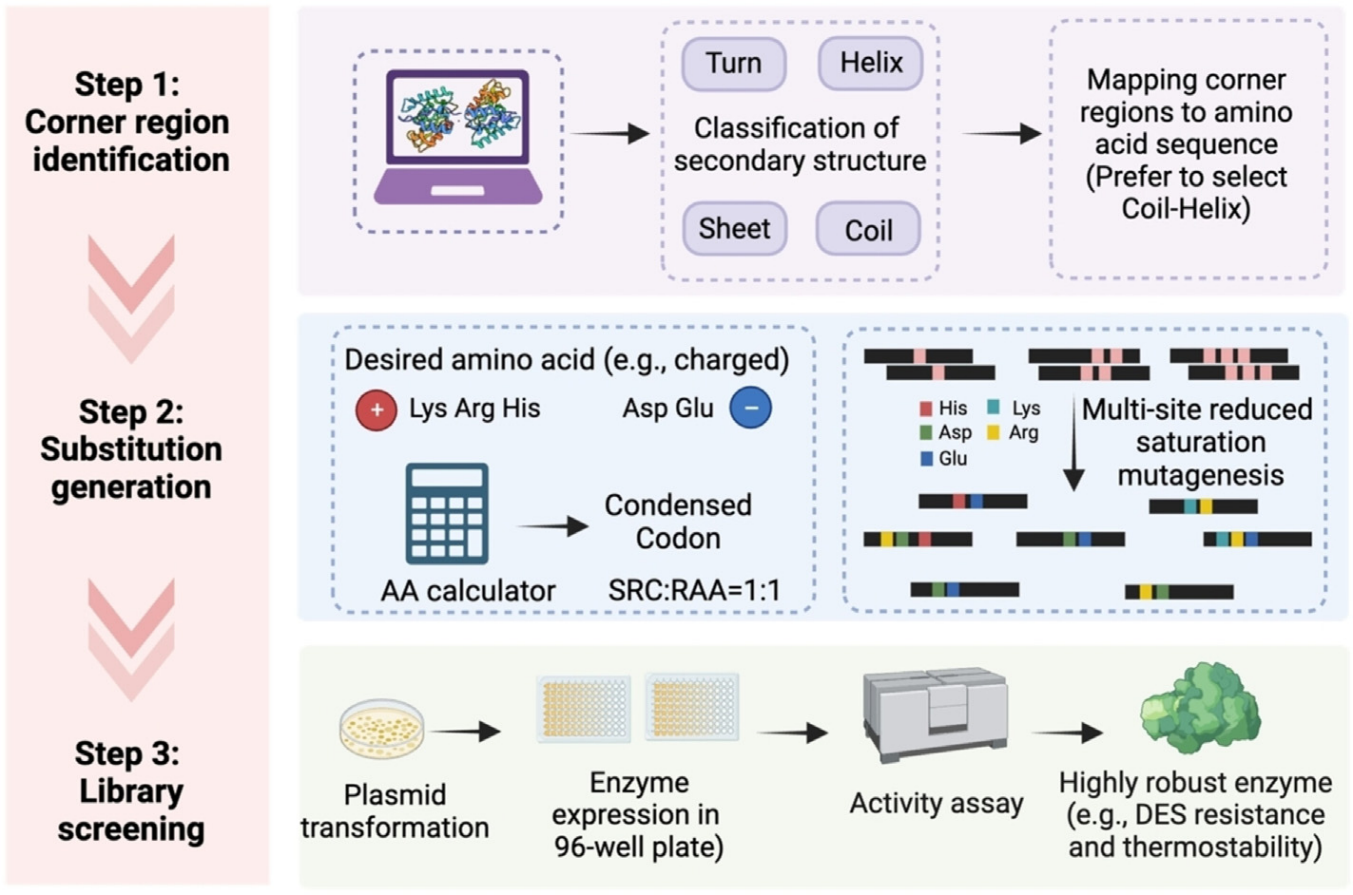
Overview of the corner engineering for screening improved variants [95]. Copyright 2024, Wiley-VCH.

Most recently, a 'corner engineering' approach, as illustrated in Fig. 10, was proposed to enhance both the solvent and thermal stability of Bacillus subtilis lipase A (BSLA) in deep eutectic solvents. The transition regions such as ω-loops between α-helix and β-sheet were proposed to play important roles in enzyme activity and stability. As a result, specific hotspots at 25 corners were targeted and charged amino acids were used to generate libraries with reduced sizes. With 2200 variants screened, several positive hits were identified locating at eight corners, and the catalytic efficiency (k_cat_/K_m_) was improved up to 10.0-fold in the DES solvents. Two additional enzymes endo- (Bs2Est) and cellulase (PvCel5A) were also successfully engineered utilizing the current strategy [95].

## 2 Conclusion

Enhancing enzyme stability remains a critical objective in enzyme engineering, underscoring the importance of employing a multifaceted approach rather than relying on a single strategy. Most studies typically adopt a combination of various methodologies, integrating traditional approaches with advanced computational techniques. Traditional methods, such as the introduction of hydrogen bonds, salt bridges, disulfide bonds, proline insertions, reduction of surface hydrophobicity, and N- and C-terminal engineering, have proven effective in enhancing enzyme stability. These established techniques provide a foundation upon which emerging methods can build [96,97].

Recent advancements in ML highlight its potential in structural prediction, rational and non-rational design, and the creation of novel enzymes. However, the availability of training datasets continues to pose challenges for broader application. Additionally, ancestral sequence reconstruction (ASR) has emerged as a valuable tool for exploring stability; yet, the reconstructed ancestral enzymes may not accurately reflect historically existing variants, introducing an element of randomness that complicates control over the resultant properties. The B-factor serves as a valuable indicator for identifying unstable residues within proteins, but the true challenge lies in modifying these sites and screening for stable mutants.

Given the limitations of these individual methods, researchers often combine B-factor analysis with other strategies to refine mutation site selection. For instance, the B-factor can be used to identify unstable residues, which are then targeted using additional methodologies to optimize stability. Similarly, ASR can serve as a starting point for directed evolution and can be effectively integrated with ML techniques to maximize improvements. Such combinations illustrate the necessity of employing multiple strategies in tandem to achieve robust and stable enzymes.

Looking ahead, as computational technologies continue to advance rapidly, it is anticipated that computational methods will play a significant role in enzyme design and modification. In this endeavor, a variety of computational tools based on different theories and algorithms have been developed to aid. For instance, tools for predicting protein stability provide researchers valuable insights for selecting beneficial mutations. While these tools may not achieve absolute precision, numerous studies have demonstrated their effectiveness in guiding mutation site selection, constructing initial mutant libraries, and screening combinatorial mutations for improved stability. The collaborative use of diverse methodologies will become the norm, driving further innovations in enzyme engineering and ensuring ongoing improvements in enzyme stability.

## Author contributions

Z.S. and Y.H. conceived the project. B.Y. and J.S. drafted the main text. All authors read and approved the final manuscript.

## Funding

This work was supported by the 10.13039/501100012166National Key Research and Development Program of China (No. 2021YFC2101900 and No. 2021YFA0910400), the 10.13039/501100001809National Natural Science Foundation of China (No. 32171462 and No. 32301277), Tianjin Synthetic Biotechnology Innovation Capacity Improvement Project (No. TSBICIP-CXRC-040), the Natural Science Foundation of Tianjin (No. 21JCJQJC00110).

## Declaration of competing interest

The authors declare that they have no known competing financial interests or personal relationships that could have appeared to influence the work reported in this paper.
